# Self‐Sorting Supramolecular Polymerization: Helical and Lamellar Aggregates of Tetra‐Bay‐Acyloxy Perylene Bisimide

**DOI:** 10.1002/anie.202006744

**Published:** 2020-08-07

**Authors:** Markus Hecht, Pawaret Leowanawat, Tabea Gerlach, Vladimir Stepanenko, Matthias Stolte, Matthias Lehmann, Frank Würthner

**Affiliations:** ^1^ Institut für Organische Chemie Am Hubland 97074 Würzburg Germany; ^2^ Center for Nanosystems Chemistry & Bavarian Polymer Institute Universität Würzburg Theodor-Boveri-Weg 97074 Würzburg Germany

**Keywords:** liquid crystals, noncovalent interactions, self-assembly, structure elucidation, supramolecular chemistry

## Abstract

A new perylene bisimide (PBI), with a fluorescence quantum yield up to unity, self‐assembles into two polymorphic supramolecular polymers. This PBI bears four solubilizing acyloxy substituents at the bay positions and is unsubstituted at the imide position, thereby allowing hydrogen‐bond‐directed self‐assembly in nonpolar solvents. The formation of the polymorphs is controlled by the cooling rate of hot monomer solutions. They show distinctive absorption profiles and morphologies and can be isolated in different polymorphic liquid‐crystalline states. The interchromophoric arrangement causing the spectral features was elucidated, revealing the formation of columnar and lamellar phases, which are formed by either homo‐ or heterochiral self‐assembly, respectively, of the atropoenantiomeric PBIs. Kinetic studies reveal a narcissistic self‐sorting process upon fast cooling, and that the transformation into the heterochiral (racemic) sheetlike self‐assemblies proceeds by dissociation via the monomeric state.

## Graphical abstract

**Cool down**: Depending on the cooling rate, a monomeric perylene bisimide equipped with four acyloxy bay substituents self‐assembles into two distinct polymorphs that exhibit distinctive optical properties and packing arrangements. The interchromophoric arrangement was elucidated in the liquid‐crystalline state, demonstrating the effect of homo‐ versus heterochiral self‐sorting, directing self‐assembly into one‐ or two‐dimensional structures, respectively.

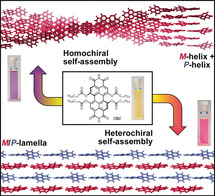

## Introduction

Perylene bisimides (PBIs) are amongst the most studied colorants in supramolecular chemistry because of their unmatched combination of favorable optical and redox properties.^1^, ^2^, ^3^, ^4^ Thus, they can afford fluorescence quantum yields close to unity^5^ and can be reduced at moderate potentials to give radical anions of high stability.^6^ Further, their high tendency to self‐assemble by π–π interactions^7^, ^8^, ^9^ in solution as well as the solid state allows construction of functional (nano)materials with tailored properties and has led to the implementation of PBIs in field‐effect transistors,^10^, ^11^, ^12^, ^13^ solar cells,^14^ and photonic devices.^15^, ^16^, ^17^ This use in different applications is supported by the ease of modification of the monomeric PBI building block, allowing fine‐tuning of the inter‐ and intramolecular interactions, as well as optical and redox properties.^18^ Particularly, substitution at the bay position with halogen,^19^, ^20^ cyano,^21^ amino,^22^ methoxy,^23^ or phenoxy substituents^24^, ^25^ is widely used to adjust the chromophore's optoelectronic features. However, the substitution at bay positions further causes a distortion of the π system because of the repulsive interactions of substituents in close proximity, resulting in a conformational chirality of these dyes.^25^ Usually, the interconversion process between the *P*‐ and *M*‐atropoenantiomers in solution is fast and separation of the two is only possible by using sufficiently large, for example, bromo substituents,^26^ by fixation of the chirality with tethers connecting the 1,7‐ and/or 6,12‐positions,^27^, ^28^ or by introduction of 2,2′‐biphenol units in 1,12‐ or 6,7‐positions.^29^ Therefore, in most cases an equilibrium between the two atropoenantiomers exists, and strongly influences the self‐assembly pathway by either homo‐ or heterochiral contacts of the chromophores.^29^ Homochiral self‐assembly leads to the formation of one‐dimensional, helical fibers of either *P*‐ or *M*‐chirality. This self‐assembly was demonstrated for a variety of PBIs which form helices in the self‐assembled state in solution^8^, ^29^, ^30^ or the columnar liquid‐crystalline phase.^31^, ^32^, ^33^ In contrast, heterochiral self‐assembly yields two‐dimensional structures of alternating *P*‐ and *M*‐atropoenantiomers, however, only observed in single‐crystals of tetra‐ and octachloro‐substituted PBIs.^34^, ^35^

**Figure 1:**
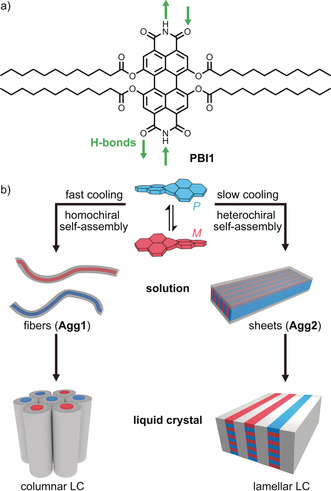
a) Chemical structure of **PBI1**. b) Schematic illustration of the cooling‐rate dependent homo‐ or heterochiral self‐assembly into fibers or sheets, respectively, and their subsequent organization into columnar or lamellar liquid crystals.

In the current study we introduce **PBI1**, a new well‐soluble PBI derivative that exhibits more similar optical properties to the parent core‐unsubstituted PBI compared to the widely applied tetraphenoxy‐substituted PBIs. **PBI1** is functionalized with four acyloxy groups at the bay position and bears free imides facilitating hydrogen‐bond (H‐bond) directed self‐assembly into supramolecular polymers (Figure 1 a). Most interestingly, **PBI1** is capable of forming both homochiral one‐dimensional fibers and heterochiral two‐dimensional sheets, depending on the cooling rate applied to a hot solution of monomers in methylcyclohexane (MCH; Figure 1 b) as determined by scanning electron microscopy (SEM) and atomic force microscopy (AFM). The two supramolecular polymorphs,^36^, ^37^ **Agg1** and **Agg2**, exhibit distinctive absorption properties originating from the unique interchromophoric arrangement, which they retain in the solid state. Polarizing optical microscopy of the polymorphs revealed their liquid‐crystalline (LC) behavior which enabled detailed studies of the supramolecular arrangement by a combination of wide‐angle X‐ray scattering (WAXS) as well as polarized UV/Vis and FT‐IR spectroscopy. The pathway complexity^38^, ^39^, ^40^, ^41^, ^42^ of the supramolecular polymerization^43^, ^44^, ^45^ that distinguishes between the two polymorphs was investigated by UV/Vis spectroscopy.

## Results and Discussion

### Synthesis

**Scheme 1:**
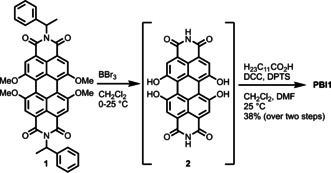
Synthesis of **PBI1** bearing four acyloxy functionalities in the 1,6,7,12‐positions.

The new **PBI1** was synthesized in a two‐step procedure starting from *N*,*N′*‐bis(1‐phenylethyl)‐1,6,7,12‐tetramethoxyperylene‐3,4:9,10‐tetracarboxylic acid bisimide (**1**) which was obtained according to a recently reported method towards tetramethoxylated PBIs (Scheme 1).^46^ This compound was dealkylated and debenzylated using boronic tribromide in dichloromethane to afford **2**. This step was followed by an esterification using dodecanoic acid under peptide coupling conditions using *N*,*N′*‐dicyclohexylcarbodiimide (DCC) and 1,4‐dimethylpyridinium *p*‐toluenesulfonate (DPTS) in a mixture of CH_2_Cl_2_ and *N*,*N*‐dimethylformamide (DMF) to obtain **PBI1** in a yield of 38 %.

### Optical Properties of the Monomeric Dye

**Figure 2:**
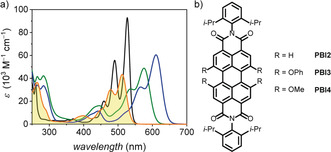
a) UV/Vis absorption spectra of **PBI1** (orange line), **PBI2** (black line), **PBI3** (green line), and **PBI4** (blue line) with *c*≈10^−5^ m in CH_2_Cl_2_. b) Chemical structures of the reference compounds **PBI2**, **PBI3**, and **PBI4**.

The yellow colored solution of monomeric **PBI1** in dichloromethane exhibits its absorption maximum at 512 nm (43 000 cm^−1^ m ^−1^), the first vibronic progression at 479 nm (30 000 cm^−1^ m ^−1^), and the S_0_‐S_2_ transition at 398 nm (7500 cm^−1^ m ^−1^; Figure 2 a). In comparison to other common PBIs, that is, **PBI2** (*λ*_max_=527 nm), **PBI3** (*λ*_max_=576 nm), and **PBI4** (*λ*_max_=610 nm; Figure 2 b), **PBI1** absorbs at lower wavelength, that is, even 15 nm hypsochromically compared to the parent **PBI2**. This blue‐shift can be explained by the electron‐withdrawing effect (−Ι‐effect) and the negligible mesomeric effect (+Μ effect) of the acyloxy bay substituents compared to the +Μ effect provided by phenoxy and methoxy in **PBI3** and **PBI4**. Similar to **PBI3** and **PBI4**, **PBI1** shows a broadened lineshape and a less pronounced vibronic fine structure. These features are characteristic for bay‐substituted PBIs and are attributed to the core‐twist induced by the steric congestion in the PBI bay‐area (see Figure S1 in the Supporting Information).^23^, ^47^

The emission properties of **PBI1** were determined in chloroform where the dye exhibits its emission maximum at 544 nm, corresponding to a small Stokes shift of 1100 cm^−1^. The fluorescence lifetime was determined to 4.8 ns which is in the expected range for monomeric PBIs (see Figure S2).^1^ **PBI1** exhibits a fluorescence quantum yield of *Φ*_Fl_=1.0 similar to **PBI2** and **PBI3**, while substitution by methoxy groups leads to a quenching of the fluorescence to *Φ*_Fl_=0.68.^46^

### Supramolecular Polymorphism

**Figure 3:**
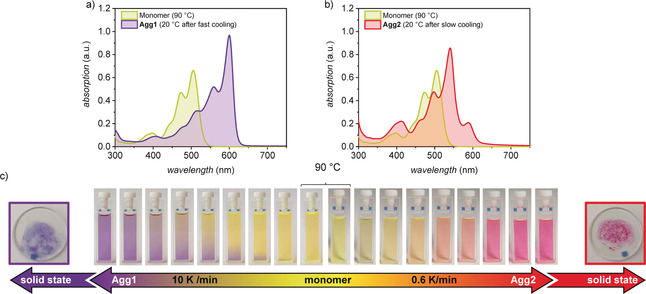
UV/Vis absorption spectra of a solution of monomers of **PBI1** (green line) at 90 °C and of the aggregates a) **Agg1** (purple line) and b) **Agg2** (red line) at *c*_0_=40 μm in MCH at 20 °C. **Agg1** and **Agg2** self‐assemble by cooling a 90 °C hot solution of **PBI1** at *c*_0_=40 μm in MCH with 10 K min^−1^ and 0.6 K min^−1^, respectively. c) Series of photographs of a solution of **PBI1** at *c*_0_=40 μm in MCH applying a cooling rate of 10 K min^−1^ to produce **Agg1** (purple) or 0.6 K min^−1^ to produce **Agg2** (red), and photographs of the respective polymorphs isolated in the solid state.

In chlorinated and aromatic solvents like dichloromethane, chloroform, tetrachloromethane, or toluene, which solubilize **PBI1** well, the dye retains its monomeric state even at higher concentrations (see Figure S3). In contrast, when dissolving **PBI1** in nonpolar aliphatic solvents like MCH (*c*_0_=40 μm), in which H‐bonds and π–π interactions can thrive,^7^ the dye self‐assembles into two different aggregates (**Agg1** and **Agg2**) depending on the cooling rate applied to a hot solution of monomers (Figure 3 c; for FT‐IR analyses, see Figure S4). Rapid cooling (10 K min^−1^) leads to the formation of **Agg1**, while slow cooling (0.6 K min^−1^) leads to the formation of **Agg2**. The polymorphs differ distinctively in their absorption profile and accordingly in color. **Agg1** shows a bathochromically shifted absorption maximum at *λ*_max_=600 nm that increases in extinction and exhibits a narrowing of the 0,0 vibronic band with a full‐width‐at‐half‐maximum (FWHM) of 300 cm^−1^ compared to the monomer with 570 cm^−1^ (Figure 3 a). These optical features are characteristic for PBI J‐aggregates and have been observed in structurally related tetra‐bay‐phenoxy substituted PBIs.^8^, ^31^, ^32^, ^48^, ^49^ In contrast, **Agg2** exhibits its main transition at 541 nm with a narrowed FWHM of 370 cm^−1^ compared to the monomer, a vibronic progression at 498 nm and a red‐shifted weaker band at 588 nm (Figure 3 b).

**Figure 4:**
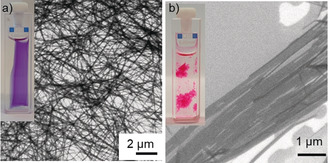
SEM images of samples of a) **Agg1** and b) **Agg2** drop‐casted onto silicon wafer (*c*_0_=40 μm, MCH). The insets show the corresponding gel‐like phase and precipitate, respectively.

At room temperature, **Agg1** transforms into a purple gel‐like phase, while **Agg2** forms a red precipitate in MCH (Figure 4). Both polymorphs can be isolated in the solid state and retain their distinctive UV/Vis absorption properties (see Figure S6). The absorption maximum of **Agg1** in the solid state can be observed at 601 nm. The FWHM of the main absorption signal increases only slightly compared to **Agg1** in solution from 300 cm^−1^ to 350 cm^−1^. The absorption maximum of **Agg2** can be observed at 538 nm and the FWHM also only slightly increases compared to the solution from 370 cm^−1^ to 470 cm^−1^. This minor increase in FWHM indicates that both polymorphs retain a highly defined intermolecular order in the solid state, enabling the elucidation of their structure by means of microscopy and scattering techniques.

### Structure Elucidation

We could investigate the morphological properties of both polymorphs by SEM of samples (MCH, *c*_0_=40 μm) drop‐casted on silicon wafer. **Agg1** shows a network of entangled fibers on the scale of several micrometers. The height of the individual fibers could be determined as 3.1±0.2 nm by AFM (see Figure S7). In contrast, **Agg2** exhibits two‐dimensional sheetlike structures with a length of several micrometers and a width of up to 1 micrometer. The height of these sheets was determined as 1.7±0.2 nm by AFM (see Figure S8). These results are in accordance with the observation of a gel‐like phase for **Agg1** and the precipitation of **Agg2**.

Both polymorphs show birefringence when investigated by polarizing optical microscopy. They can be aligned by mechanical shearing, indicating the fluid nature of the material originating in the nanosegregation of the rigid π‐core and the flexible alkyl chains (see Figure S9). Accordingly, we were able to investigate the supramolecular assemblies by WAXS experiments of aligned fibers to elucidate the respective intermolecular arrangement. Both polymorphs were prepared on a 10 mg scale by applying the required cooling rates to hot solutions of **PBI1** in MCH (*c*_0_=40 μm), and were isolated by centrifugation and dried under reduced pressure. The resulting LC materials were subsequently aligned by fiber extrusion at ambient temperature from their LC states.

**Figure 5:**
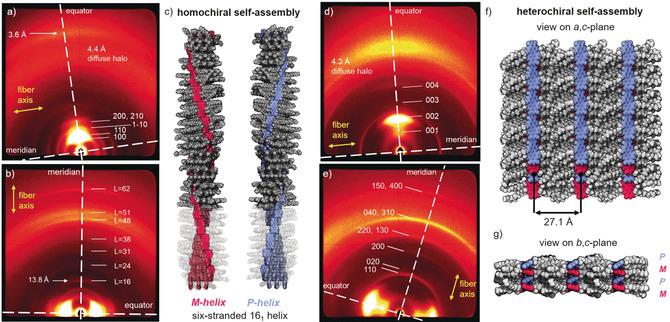
WAXS diffraction patterns of a a,d) lying and b,e) standing fiber of a,b) **Agg1** and d,e) **Agg2** at 25 °C. Yellow arrows indicate the relative orientation of the fibers and the meridian and equator are highlighted with white dashed lines. Layer lines in b) are indexed with L. Note: Meridian and equator are defined here with respect to the fiber direction to enable a consistent discussion. c) Homochiral self‐assembled structure of the six‐stranded 16_1_‐helix of **Agg1** in the respective *M*‐ (red) or *P*‐helix (blue). Parts of the alkyl chains are depicted semitransparent to illustrate the six PBI strands. Heterochiral self‐assembled structure of the 2D sheets of **Agg2** which arrange in a lamellar lattice with view on the f) *a*,*c*‐plane and g) *b*,*c*‐plane. *M*‐ and *P*‐atropoenantiomers of **PBI1** are colored red and blue, respectively.

The WAXS pattern of a lying fiber of **Agg1** shows equatorial reflections which can be indexed according to a columnar rhombohedral lattice (Col_rhomb_) with *a=*28.8 Å and *γ*=78.8° (Figure 5 a). The diffuse halo at 4.4 Å corresponds to the liquid‐like alkyl chains. The X‐ray diffraction pattern of a standing fiber of **Agg1** shows meridional and off‐meridional reflections that indicate a periodic organization of the chromophores along the column (Figure 5 b). The first meridional signal corresponds to the length of the axial translation subunit and can be observed at 13.8 Å, which is the size of the PBI chromophore along the long axis.^31^, ^32^, ^33^ This data indicates that the PBIs are oriented parallel to the columnar long axis forming H‐bonded strands, and is supported by polarized FT‐IR (see Figure S10 a) and UV/Vis spectroscopy (see Figure S11 a) of shear‐aligned thin films, which reveal that NH stretching vibrations in H‐bonds and the S_0_–S_1_ transition of the PBIs are aligned parallel to the shearing and column direction. This result is further confirmed by a diffuse signal at 3.6 Å on the equator, which is characteristic for such PBI assemblies and originates in the π–π stacking of the chromophores perpendicular to the columnar long‐axis.^31^, ^32^, ^33^ The meridional signal at 13.8 Å can be indexed as layer line L=16 of a helical arrangement and consequently all other diffuse meridional and off‐meridional signals are positioned at layer lines L=24, 31, 38, 48, 51 and L=62. Accordingly, 16 molecules form the helical repeat of 16×13.8 Å=220.8 Å. The correlation length of the equatorial signal at 3.6 Å amounts to six molecules (see Supporting Information). This value implies that about six strands of H‐bonded PBIs form a column and is supported by the reasonable density of 1.05 g cm^−1^ when the columnar stratum is filled by the integer number of six molecules (for details see the Supporting Information). Therefore, **Agg1** is composed of a sextuple‐stranded 16_1_ helix with a 22.5° twist per molecule. Such a helical arrangement can only be formed by homochiral self‐assembly leading to the formation of either *P*‐ or *M*‐helices. As **PBI1** is achiral, both *M*‐ and *P*‐helical self‐assemblies coexist in the columnar liquid crystal. The individual H‐bonded strands are longitudinally displaced by about 7 Å, leading to a slipped‐stack arrangement causing the absorption spectrum which is typical for PBI J‐aggregates.^8^, ^31^, ^32^, ^34^

The self‐assembled structure was modelled with the program *Accelrys Materials Studio 2017 R2*. Accordingly, core‐twisted PBIs with the same axial chirality were arranged to form H‐bonded strands in a slipped‐stack in the sextuple‐stranded helix (Figure 5 c, for details see the Supporting Information). The helix was optimized in the rhombohedral unit cell with the force field COMPASS II, applying the Ewald summation method until the nonbonding energy was strongly negative. With the optimized structure, we could simulate the fiber diffraction pattern with the program CLEARER.^50^ The simulated pattern is in good agreement with the experiment showing the essential signals corresponding to the Col_rhomb_ lattice as well as the meridional signal at 13.8 Å (see Figure S13 a).

The related analysis of the WAXS pattern of a lying fiber of **Agg2** shows four equidistant reflections that could be indexed as 001, 002, 003, and 004 signals of a lamellar lattice (*c=*27.1 Å; Figure 5 d). However, these signals are very broad, indicating a weak correlation between the lamellae. Accordingly, the correlation length could be calculated to be only two lamellae (for details see Supporting Information). In contrast, the meridional signals related to the intralamellar arrangement of the PBIs are well defined (Figure 5 e). They can be apparently indexed according to a rectangular centered lattice (*a=*14.1 Å, *b=*18.2 Å). The corresponding unit cell comprises four PBI molecules assuming a reasonable density of 1.11 g cm^−3^. The PBIs are oriented in parallel with the layer direction, that is, perpendicular to the *c*‐axis as determined with polarized FT‐IR (see Figure S10 b) and UV/Vis spectroscopy (see Figure S11 b).

To rationalize the absorption spectra of **Agg2** and thus gain further information on the arrangement of the dyes within the unit cell, an interplay between short‐ and long‐range coupling has to be taken into account.^51^, ^52^ The latter arises from the interaction of the transition dipole moments as described within the conventional Kasha exciton theory,^53^ whereas the short‐range coupling is caused by the HOMO–HOMO and LUMO–LUMO overlap of the π‐stacked chromophores, and is very sensitive to structural arrangements.^52^, ^54^ Therefore, small changes of the longitudinal shift of the PBI chromophores can lead to distinctly different absorption spectra, enabling one to derive information on the chromophore arrangements.^52^, ^55^ The absorption spectrum of **Agg2** is in very good agreement with the calculated spectrum for π‐stacked perylene dyes exhibiting a longitudinal shift of about 5 Å as reported by Hestand and Spano (see Figure S15).^52^ Since the HOMO and LUMO distribution of perylene^53^ and PBI^1^ chromophores are almost identical, we can use the perylene spectra as reference. Accordingly, the spectral signature of **Agg2** results from the interference of long‐range and short‐range coupling in the so‐called resonant regime (i.e. the Frenkel and charge‐transfer state are of similar energy)^51^ and shows one intense absorption peak at 539 nm and less intense absorption bands at higher and lower energies (for a detailed discussion see the Supporting Information).

With the help of the exciton‐vibrational spectral pattern analysis, the unit cell of LC **Agg2** was generated with PBI chromophores that are longitudinally shifted by 5 Å (see Figure S12). This special in‐plane shift, however, breaks the first assigned centered symmetry of the four strands and the planar unit cell must be primitive. Thus the absence of reflection with h+k=2*n*+1 is accidental, and the cell is apparently only pseudocentered (see the Supporting Information). Figures 5 f and g show a supercell of this arrangement that highlights the alternating arrangement of *P*‐ and *M*‐atropoenantiomers. Using the constructed unit cell, the diffraction pattern was simulated using CLEARER, which confirms the absence of the most prominent reflections for the primitive unit cell (see Figure S13 b).^50^

### Pathway Complexity of the Supramolecular Polymerization

**Figure 6:**
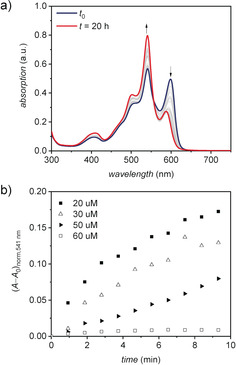
a) Time‐dependent UV/Vis absorption spectra of a 1:1 mixture of **Agg1** and **Agg2** (40 μm in MCH) at 35 °C. b) Time course of the transformation from **Agg1** to **Agg2** at 35 °C in dependence of the concentration *c*_0_ of **Agg1** in MCH.

Lastly, we were interested in a more detailed investigation of the supramolecular polymerization and the underlying pathway complexity that leads to the formation of the respective homochiral one‐dimensional and heterochiral two‐dimensional polymorphs. Temperature‐dependent UV/Vis spectroscopy of **PBI1** (*c*_0_=40 μm, MCH) showed that the self‐assembly of both, **Agg1** (cooling rate 10 K min^−1^) and **Agg2** (cooling rate 0.6 K min^−1^), follow a cooperative nucleation‐elongation mechanism (see Figure S17). This mechanism is reasonable as more than one intermolecular force, namely π–π interactions and H‐bonding, contribute to the formation of the supramolecular polymers. When mixing **Agg1** and **Agg2** in a 1:1 ratio (*c*_0_=40 μm, MCH), the UV/Vis absorption spectrum shows a superposition of the individual spectra (blue line, Figure 6 a). Time‐dependent experiments at 35 °C revealed an interconversion of **Agg1** into **Agg2** as indicated by the decreasing absorption at 600 nm and the concomitant shift to 588 nm as well as the increasing absorption at 541 nm (Figure 6 a). This interconversion suggests that **Agg2** is the thermodynamically favored product, while **Agg1** is formed under kinetic control. The pathway‐complexity can be probed by time‐dependent UV/Vis experiments at different concentrations.^38^, ^39^ Accordingly, the conversion of **Agg1** into **Agg2** was followed at a range of concentrations from *c*_0_=20 μm to *c*_0_=60 μm in MCH at 35 °C (see Figure S18). At *c*_0_=60 μm, no significant spectral change can be observed over a period of 10 minutes. However, at lower concentrations the absorption signal at 600 nm corresponding to **Agg1** decreases in intensity whilst the new absorption band at 541 nm corresponding to **Agg2** arises. This change can be followed by the change in absorption at 541 nm over time, thereby demonstrating an increasing rate with decreasing concentration (Figure 6 b, see Figure S18). These results indicate that **Agg1** is an off‐pathway kinetic product and that the interconversion of **Agg1** into **Agg2** takes place by the fully dissociated monomeric state and is therefore favored at lower concentrations where the concentration of available monomer is higher.^38^, ^39^ Putting these results into perspective, it is reasonable to assume that the initial dimer pair formation, that is, homochiral (*M*/*M* or *P*/*P*) or heterochiral (*P*/*M* or *M*/*P*) determines the outcome of the self‐assembly pathway and thereby the final helical fiber or sheet‐type morphology. **Agg1** is formed by a homochiral assembly of either only *P*‐ or only *M*‐atropisomers, while the formation of **Agg2** requires an alternating, heterochiral self‐assembly. Apparently, once *P*‐ or *M*‐chirality is established within the supramolecular structure of the kinetic product **Agg1**, the fibers cannot come unwound to form the heterochiral, sheetlike structure **Agg2** even though it is the thermodynamically favored product. The pathway via the monomeric state, however, enables the structural transformation because of the fast interconversion between the two atropoenantiomers in solution. It is noteworthy that the herein elucidated narcissistic versus social self‐sorting^56^ in one‐ and two‐dimensional supramolecular polymerization into **Agg1** and **Agg2** under either kinetic or thermodynamic control directly relates to the formation of conglomerate and racemate crystals in three‐dimensional self‐assembly. Unfortunately, the conversion of the kinetic polymorph **Agg1** into the thermodynamic polymorph **Agg2** by a seeded^37^, ^57^ supramolecular polymerization approach could not be achieved in a satisfactory manner as even at high seed ratios of **Agg2** only a minor transformation into the thermodynamically favored polymorph was observed (see Figure S19).

## Conclusion

In summary, we presented a new **PBI1** dye bearing four acyloxy substituents at bay positions. In this study, we showed that **PBI1** forms two polymorphs upon cooling a hot solution in methylcyclohexane, depending on the applied cooling rate. The two polymorphs show distinctive absorption profiles in solution and in the liquid‐crystalline state, which could be related to a difference in the longitudinal shift of the H‐bonded strands in the self‐assembled structures. The interchromophoric arrangement in the respective one‐ and two‐dimensional polymorph could be elucidated by polarized spectroscopy and X‐ray scattering revealing the formation of a columnar (**Agg1**) and a lamellar structure (**Agg2**). Based on these results we were able to propose packing models for both polymorphs. While the helical structure of **Agg1** is formed by a homochiral arrangement of the respective *P*‐ and *M*‐atropoenantiomers, the sheets of **Agg2** are formed by an alternating heterochiral arrangement of the two. Accordingly, formation of **Agg1** and **Agg2** are particularly illustrative examples of conglomerate versus racemic self‐sorting phenomena. Time‐dependent UV/Vis spectroscopy in solution indicated that **Agg2** whose formation is initiated from heterochiral dimer pairs is the thermodynamically favored product while **Agg1** is formed in a kinetic process from homochiral dimer pairs in an off‐pathway mechanism. Accordingly, **PBI1** provided unprecedented insights into self‐assembly pathways from the monomer via aggregates in solution up to the bulk liquid‐crystalline state. Given their interesting (opto)electronic properties, **PBI1** or its derivatives might also be interesting candidates for future applications in photovoltaic and photonic devices.

## Conflict of interest

The authors declare no conflict of interest.

## Supporting information

As a service to our authors and readers, this journal provides supporting information supplied by the authors. Such materials are peer reviewed and may be re‐organized for online delivery, but are not copy‐edited or typeset. Technical support issues arising from supporting information (other than missing files) should be addressed to the authors.

Supplementary
