# Supplementary material for: Self‐Sorting Supramolecular Polymerization: Helical and Lamellar Aggregates of Tetra‐Bay‐Acyloxy Perylene Bisimide
Source: Angew Chem Int Ed Engl. 2020 Aug 7;59(39):17084–90. doi: 10.1002/anie.202006744 (PMC7540443; doi:10.1002/anie.202006744)
Supplement: Supplementary file 1 — Supplementary [file ANIE-59-17084-s001.pdf]

Angewandte Chemie
Eine Zeitschrift der Gesellschaft Deutscher Chemiker

Supporting Information

Self-Sorting Supramolecular Polymerization: Helical and Lamellar Aggregates of Tetra-Bay-Acyloxy Perylene Bisimide

Markus Hecht, Pawaret Leowanawat, Tabea Gerlach, Vladimir Stepanenko, Matthias Stolte, Matthias Lehmann, and Frank Würthner**

anie_202006744_sm_miscellaneous_information.pdf

Table of contents

1. Materials and Methods 2
2. Synthesis 5
3. DFT calculations 6
4. Absorption and fluorescence spectroscopy 7
5. Solvent dependent UV/Vis absorption of **PBI1** 8
6. FT-IR spectroscopy in solution 9
7. Cyclic and square wave voltammetry 10
8. UV/Vis spectroscopy of thin films 11
9. Atomic force microscopy 12
10. Polarizing optical microscopy 13
11. Polarized spectroscopy of aligned thin films 14
12. Molecular modelling 15
13. X-ray scattering 18
14. Comparison to calculated spectra 20
15. Temperature-dependent UV/Vis absorption studies 22
16. Time-dependent UV/Vis spectroscopy 23
17. Seed-induced supramolecular polymerization 24
18. NMR spectra of **PBI1** 25
19. Supporting References 26

1. Materials and Methods

Materials

Reagents were purchased from commercial suppliers and used as received without further purification. *N*,*N*'-Dicyclohexylcarbodiimide (DCC) was freshly distilled prior to its use. Solvents were distilled and dried by standard procedures. All reactions were carried out under nitrogen atmosphere.

Column chromatography

Column chromatography was performed with commercial glass columns using silica gel 60M (particle size 0.04-0.063 mm; Macherey-Nagel GmbH & Co. KG, Germany) as stationary phase.

NMR spectroscopy

^{1}H and ^{13}C nuclear magnetic resonance (NMR) spectra were recorded on a Avance-400 spectrometer (Bruker-Daltonics GmbH, Germany) operating at 400 MHz (^{1}H) or 100 MHz (^{13}C), with the residual protic solvent used as the internal standard. The chemical shifts (δ) are reported in parts per million (ppm). Multiplicities for proton signals are abbreviated as s and m for singlet and multiplet, respectively.

High resolution mass spectrometry

High resolution mass spectra (HRMS) were recorded on an ESI micrOTOF focus spectrometer (Bruker Daltonic GmbH, Germany).

UV/Vis absorption spectroscopy in solution

UV/Vis absorption spectra in solution were recorded using a V-770 spectrophotometer (JASCO Inc., Japan). The spectra were measured in quartz glass cuvettes using spectroscopic grade solvents. Temperature control was accomplished by a PAC-743R Peltier system (JASCO Inc., Japan). Extinction coefficients were calculated from Lambert-Beer's law.

Fluorescence spectroscopy in solution

Fluorescence spectroscopy in solution was carried out with an FLS980 spectrometer (Edinburgh Instruments Ltd., UK) by optical dilution method (OD_{max} < 0.05). The fluorescence quantum yield of **PBI1** in chloroform was determined as average value of four different excitation wavelengths (455, 460, 465 and 470 nm) using *N*,*N*'-bis(2,6-diisopropylphenyl)-3,4,9,10-perylenetetracarboxylic diimide as reference.[S1] The signals were corrected for the different refractive indices according to common procedure. Time-resolved measurements

were performed with a ps laser diode and a TCSPC detection unit

Cyclic voltammetry and square wave voltammetry

Cyclic voltammetry and square wave voltammetry was carried out in a three electrode single-compartment cell on a standard electrochemical analyser (EC epsilon, BAS Instruments, UK). A glassy carbon disc electrode was used as a working electrode, a platinum wire as a counter electrode and an Ag/AgCl reference electrode using ferrocene/ferrocenium (Fc/Fc^{+}) as an internal standard for the calibration of the potential. The measurements were carried out under argon atmosphere in dichloromethane and tetrabutylammonium hexafluorophosphate (recrystallized from ethanol/water and dried under vacuum) was added as supporting electrolyte.

Scanning electron microscopy

SEM images were recorded using a Zeiss Ultra Plus field emission scanning electron microscope equipped with GEMINI e-Beam column operated at 1.5 kV with an aperture size set to 30 µm to avoid excessive charging and radiation damage of the areas imaged. The sample was prepared by drop-casting of the sample solution in MCH (40 µM) onto silicon wafer.

Atomic force microscopy

AFM measurements were performed under ambient conditions using a Bruker Multimode 8 SPM system operating in tapping mode in air. Silicon cantilevers (OMCL-AC200TS, Olympus) with a resonance frequency of ~150 kHz and a spring constant of ~10 Nm^{-1} were used.

UV/Vis absorption spectroscopy of thin films

UV/Vis absorption spectroscopy of thin films was carried out with an Axio Imager 2 (Carl Zeiss AG, Germany) polarizing optical microscope equipped with a cooled CCD spectrometer MCS-CCD PCI (Carl Zeiss AG, Germany).

FT-IR spectroscopy

FT-IR spectra were recorded with an AIM-8800 infrared microscope connected to a IRAffinity FT-IR spectrometer (Shimadzu Corp., Japan). The sample was prepared as a thin-film on a KBr substrate (thickness 2mm). Polarization was achieved by using a precision automated polarizer (ZnSe) (PIKE Technologies, USA). This includes the PIKE Technologies Motion Control Unit and AutoPro software.

Polarized optical microscopy

The liquid-crystalline materials were examined under an Axio Imager 2 (Carl Zeiss AG, Germany) polarizing optical microscope.

Wide-angle X-ray scattering

WAXS measurements were performed on a Bruker Nanostar (Detector Vantec2000, Microfocus copper anode X-ray tube Incoatec). Liquid-crystalline samples were prepared by fiber extrusion using a mini-extruder. The measurements were carried out in Mark capillaries (Hilgenberg) positioned perpendicular to the incident X-ray beam. WAXS experiments were performed at a sample-detector distance of 21 cm, with the detector tilted by 14° upwards in order to investigate the angular range of 2θ = 0.8°-28°. This allowed only the investigation of a section of the XRS pattern, therefore samples with lying and standing extruded fibers have been prepared to study the equatorial and the meridional signals in detail. Lying fiber means horizontal oriented fiber with respect to the tilt direction of the detector, while standing fiber means parallel oriented fiber with respect to the tilt direction of the detector. Silver behenate was used as calibration standard for WAXS. All X-ray data were processed and evaluated with the program datasqueeze.

Molecular modelling

The molecular models were created with Dassault Systèmes BIOVIA, Materials Studio 2017 R2 (San Diego, USA).

2. Synthesis

Synthesis of 1,6,7,12-tetrahydroxyperylene-3,4:9,10-bis(dicarboximide) (2)

Into a solution of **1** (30.0 mg, 42 μmol) in dry CH_2Cl_2 (5 mL) was added BBr_3 (210 mg, 838 μmol, 20 eq.) in dry CH_2Cl_2 (3 mL) dropwise at 0 °C. The reaction mixture was allowed to stir at 0 °C for 1 h and at 25 °C for 12 h. The solvent was removed by distillation and MeOH:H_2O (1:4) was added slowly to quench the reaction. The solid suspension was sonicated for 30 min followed by filtration and drying in vacuum to give **2** as a dark blue solid which was used for the next step without further purification.

Synthesis of PBI1

DCC (60 mg, 0.29 mmol, 5.3 eq.) in CH_2Cl_2 (0.2 ml) was added to a mixture of **2** (25 mg, 49 μmol), dodecanoic acid (50 mg, 0.25 mmol, 4.5 eq.) and DPTS (30 mg, 0.11 mmol, 2.0 eq.) in DMF:CH_2Cl_2 (2:1, 0.3 ml). The reaction mixture was stirred at room temperature for 48 h. The solution was then concentrated and the solid residue was purified by column chromatography (silica gel, CH_2Cl_2: Et_2O, 100:0 to 98:2), followed by precipitation in cold MeOH to give **PBI1**.

Yield: 22.3 mg (38%) of a purple solid.

^1H NMR (400 MHz, $CDCl_3$, ppm): δ = 8.67 (s, 2H), 8.45 (s, 4H), 2.60 (m, 8H), 1.69 (m, 8H), 1.44-1.18 (m, 64H), 0.88 (m, 12H). ^{13}C NMR (100 MHz, $CDCl_3$, ppm) δ = 171.3, 162.4, 148.5, 131.2, 126.4, 124.1, 123.8, 123.6, 34.4, 32.1, 29.9, 29.8, 29.6, 29.5, 29.4, 29.2, 24.8, 22.8, 14.3. HRMS (ESI, acetonitrile/chloroform 1:1, pos. mode): m/z calcd for $C_{72}H_{98}N_2NaO_{12}$: 1205.7012 $[M+Na]^+$, found: 1205.6984. m.p. = 230 °C. UV/Vis ($CHCl_3$): λ_{max} [nm] (ε [M^{-1} cm^{-1}]) = 513 (40000), 481 (26000). Φ_{Fl} ($CHCl_3$)= 1.0. CV (CH_2Cl_2, 0.1 M TBAHFP, vs. Fc/Fc^+): E_{red1} = –1.27 V, E_{red2} = –1.53 V.

3. DFT calculations

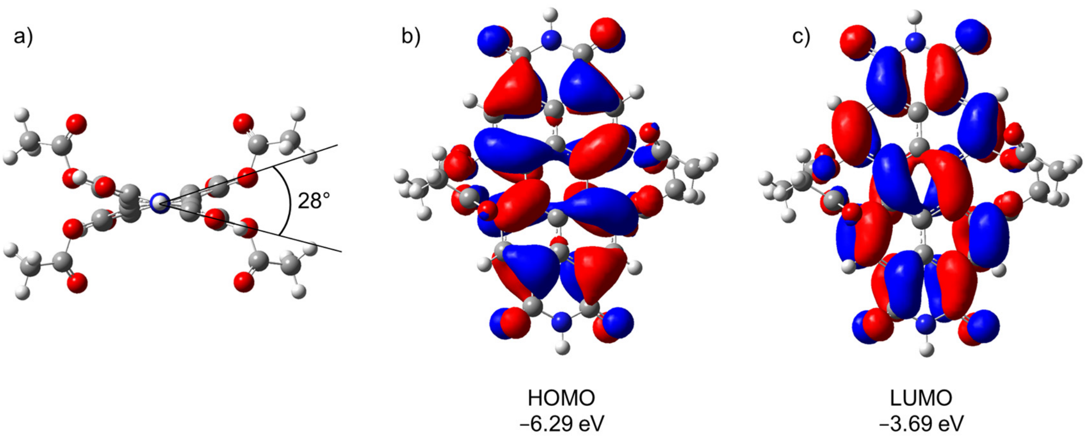

Figure S1. a) Geometry optimized structure of **PBI1** by DFT (RB3LYP/dev2-SVP) in the direction along the long molecular axis to display the torsion between the two naphthalene units of the molecule (the undecyl chains were truncated to methyl groups) and b) HOMO and c) LUMO levels of **PBI1**.

4. Absorption and fluorescence spectroscopy

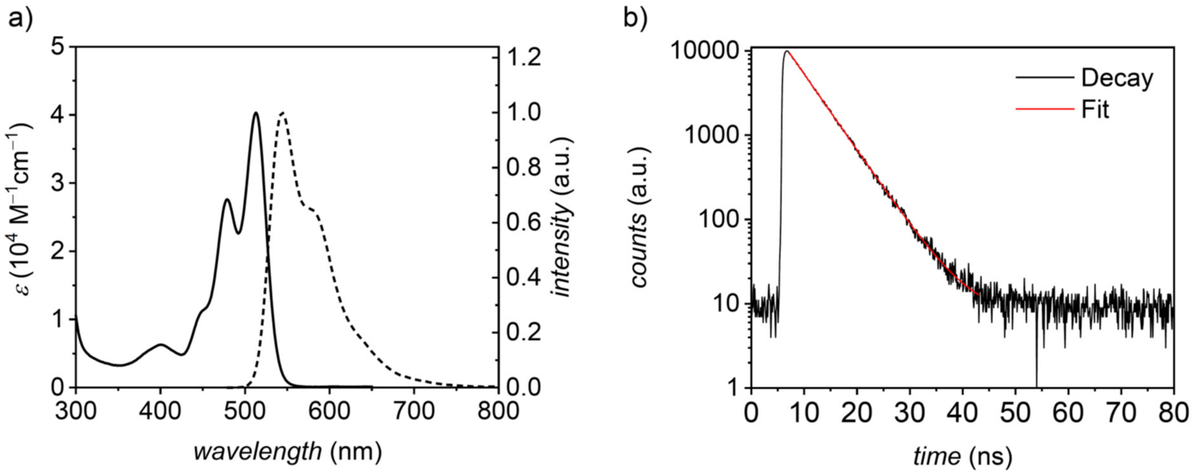

Figure S2. a) UV/Vis absorption (solid line) and normalized fluorescence (dashed line) spectra of **PBI1** in $CHCl_3$ (c_0 = 9.1 × 10^{-7} M) at 20 °C. b) Fluorescence lifetime measurement of **PBI1** in $CHCl_3$ (c_0 = 9.1 × 10^{-7} M, λ_{ex} = 485 nm) at 20 °C as well as the monoexponential fit (red, τ = 4.8 ns) of the decay.

5. Solvent dependent UV/Vis absorption of PBI1

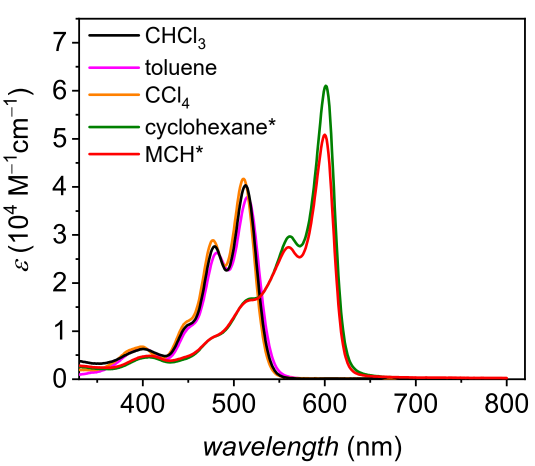

Figure S3. Solvent-dependent absorption spectra of **PBI1** in $CHCl_3$ (black), toluene (pink), CCl_4 (orange), cyclohexane (green) and methylcyclohexane (red) at c_0 = 40 µM and 22 °C. Solutions in cyclohexane and MCH were prepared by heating the solution to dissolve the material and letting it rapidly cool down at ambient conditions. This fast cooling process leads to the formation of the kinetic product **Agg1**. *Please note: in non-polar solvents, the formed aggregate tends to partially separate from the solvent rendering the extinction coefficient error-prone.

6. FT-IR spectroscopy in solution

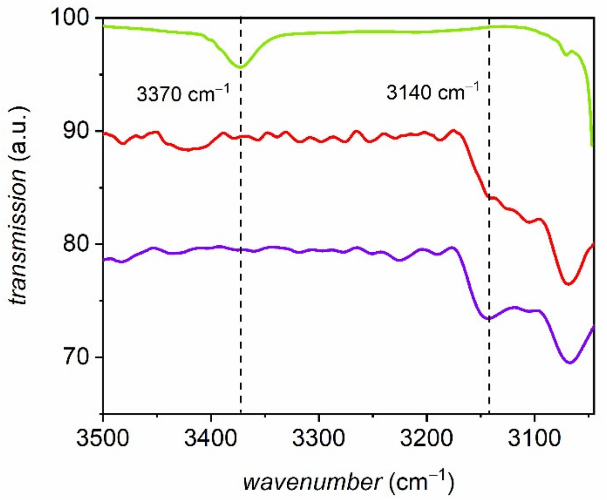

Figure S4. FT-IR spectra of solutions of monomeric **PBI1** in $CHCl_3$ (c = 200 µM, green), **Agg1** in MCH (c = 200 µM, purple) and **Agg2** in MCH (c = 200 µM, red).

The involvement of intermolecular H-bonds in the supramolecular polymerization was investigated with FT-IR spectroscopy. The FT-IR spectra of **Agg1** (purple) and **Agg2** (red) display a N-H stretching signal at 3140 cm^{-1} which is shifted to lower energies compared to the N-H stretching signal of monomeric **PBI1** in chloroform (3370 cm^{-1}), clearly demonstrating the presence of H-bonds in **Agg1** and **Agg2**.

7. Cyclic and square wave voltammetry

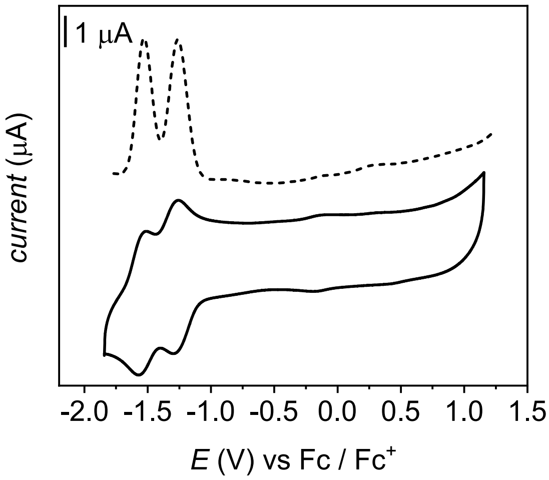

Figure S5. Cyclic (solid line) and square wave voltammetry traces (dashed line) of **PBI1**. The measurements were performed in dichloromethane ($c_0 \approx 1 \times 10^{-4}$ M) at room temperature with tetrabutylammonium hexafluorophosphate (0.1 M) as electrolyte (scan rate: 100 mV s^{-1}).

The change in electronic properties by the introduction of the four ester functionalities in bay-position was investigated by cyclic and square wave voltammetry. These measurements showed two reversible reductions at –1270 mV and –1530 mV *vs*. Fc/Fc^+ to the mono- and dianion, respectively (see Figure S5). No oxidation could be observed at potentials up to 1000 mV. Considering the optical bandgap of **PBI1** in dichloromethane, the HOMO and LUMO levels can be estimated as –6.30 eV and –3.88 eV, respectively, with the energy level of Fc/Fc^+ set to –5.15 eV *vs.* vacuum.[S2] These values are in good agreement with those obtained by DFT calculations (see Figure S1).

8. UV/Vis spectroscopy of thin films

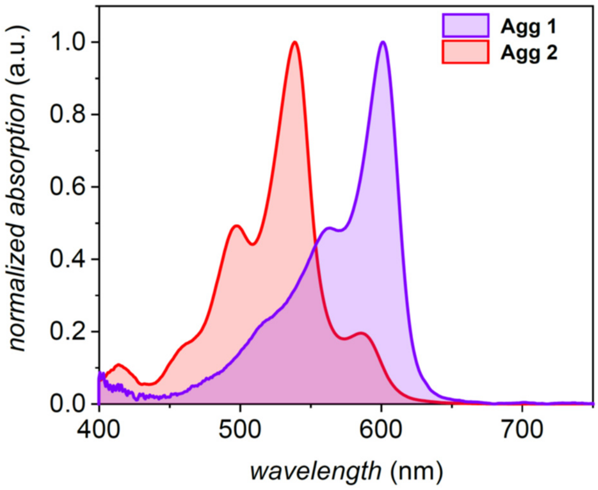

Figure S6. Normalized UV/Vis absorption spectra of drop-casted thin films of **Agg1** (purple) and **Agg2** (red) on quartz at 25 °C.

9. Atomic force microscopy

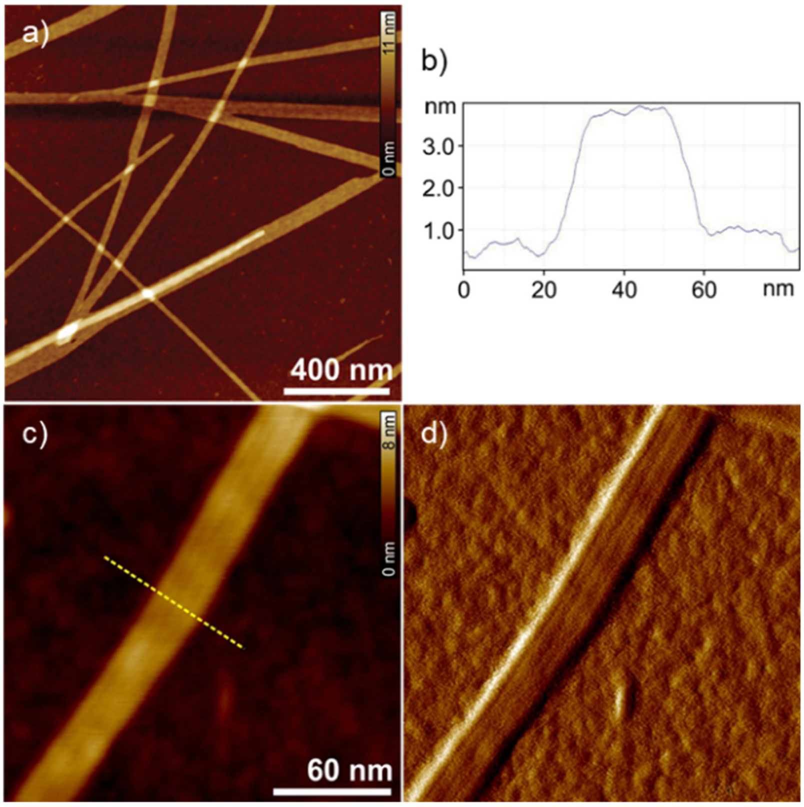

Figure S7. a,c) AFM height images of **Agg1** prepared by spin-coating a solution in MCH (c_0 = 40 µM) onto silicon wafer. b) Cross-section analysis along the yellow dashed line in c). d) Corresponding phase image of c) highlighting the individual fibers within the bundle.

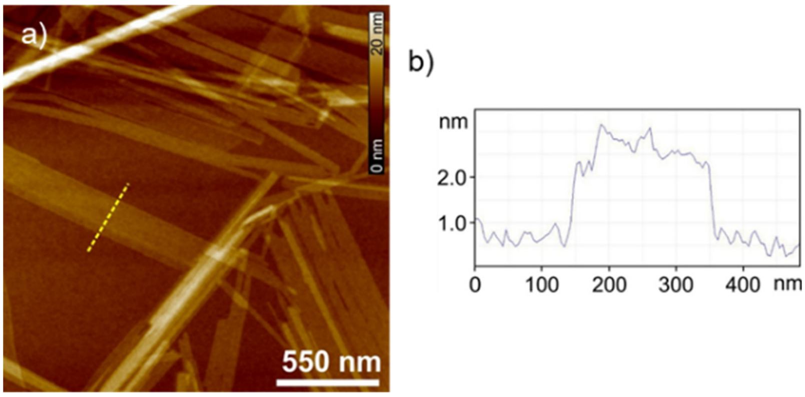

Figure S8. a) AFM height image of **Agg2** prepared by drop-casting a solution in MCH (c_0 = 40 µM) onto silicon wafer. b) Cross-section analysis along the yellow dashed line in a).

10. Polarizing optical microscopy

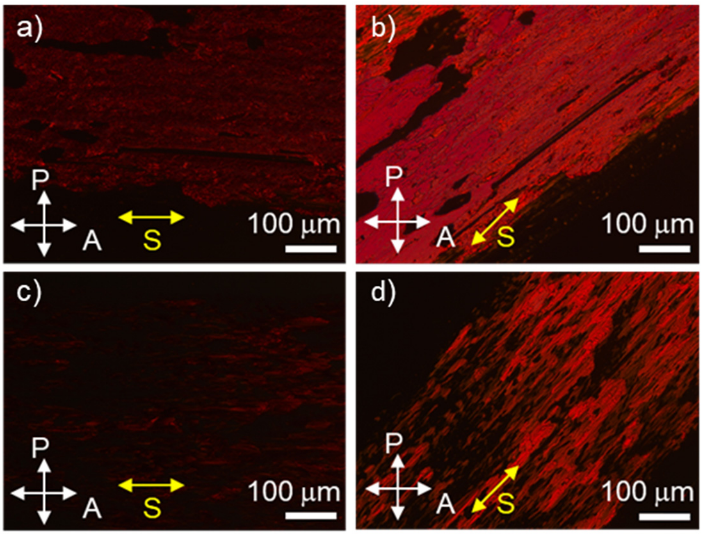

Figure S9. Polarizing optical microscopy images of shear-aligned thin films of a,b) **Agg1** and c,d) **Agg2** with crossed polarizer (P) and analyzer (A) and the shearing direction (S) a,c) parallel or b,d) in a 45°rotational displacement to P and A.

11. Polarized spectroscopy of aligned thin films

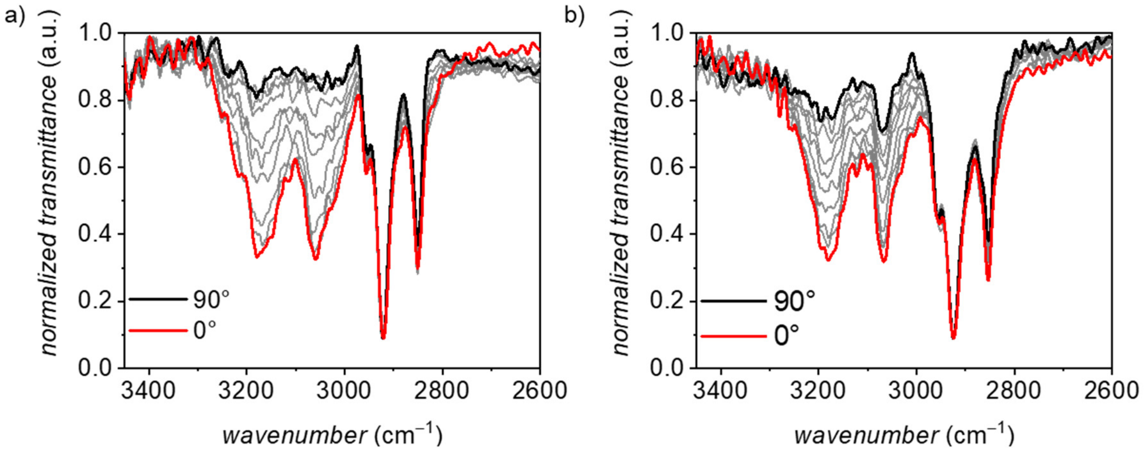

Figure S10. Polarized FT-IR spectra with different polarization angles of an aligned sample of a) **Agg1** and b) **Agg2**. The sample was sheared using an extruded fiber for friction transfer on a KBr substrate. The black and red line show the spectra for the polarizer orientated perpendicular and parallel to the shearing direction, respectively.

The polarized FT-IR measurements show that the NH stretching vibration at 3180 cm^{-1} corresponding to the hydrogen-bonded imide units is maximized when measuring along the shearing direction (red) while it practically disappears when measuring perpendicular to the shearing direction (black). This indicates that the PBIs are oriented with their long axis along the shearing direction and therefore a) along the columnar long axis and b) perpendicular to the lamellar *c*-axis.

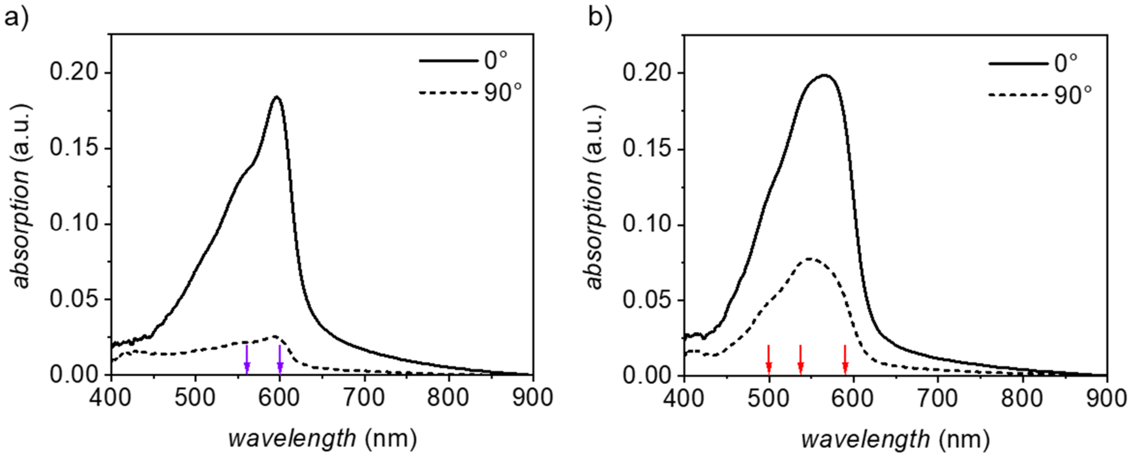

Figure S11. UV/Vis absorption spectra of a shear-aligned thin film of a) **Agg1** and b) **Agg2** on quartz measured with linearly polarized light parallel (solid line) and perpendicular (dashed line) to the shearing direction. Arrows indicate the position of absorption maxima in drop-casted samples (see Figure S6).

The polarized UV/Vis absorption spectra show that the PBI main transition dipole moment (S_0-S_1 transition) is oriented parallel to the shearing direction for both, **Agg1** and **Agg2**. Upon shearing, absorption signals get broadened as mechanical shearing partially disrupts the interchromophoric arrangement.

12. Molecular modelling

The number of molecules Z in a unit cell can be calculated using equation S1:

$$Z = \frac{\rho * N_\mathrm{A} * V_\mathrm{unit\,cell}}{M}, \tag{S1}$$

Where ρ is the density, N_A the Avogadro constant, $V_\mathrm{unit\,cell}$ the volume of the unit cell and M the molecular weight.

In case of **Agg1**, equation S2 can be used to calculate the volume of the unit cell:

$$V_\mathrm{unit\,cell} = V_\mathrm{col-strat} = A_{rhombus} * h. \tag{S2}$$

Where A_rhombus is the area of the rhombus and h the height of the columnar stratum. The height can be in principle chosen arbitrarily, however, since the axial translational subunit is 13.8 Å it has been naturally chosen as stratum height. Accordingly, using equation S1, filling the unit cell with six molecules would require a reasonable density of ρ = 1.05 g cm^{-1}.

The model of self-assembled **Agg1** is based on π-stacked hexamers. The hexameric units were built by co-facial packing arrangement of the chromophores in a π-π-stacking distance of 3.6 Å. The PBIs are longitudinally and rotationally displaced by 6.9 Å and 11.5°, respectively. These hexameric units were then translationally positioned at a distance of 13.8 Å rotated by 22.5° to form one helical column consisting of 96 molecules. This six-stranded assembly was placed in a rhombohedral unit cell (a = 28.8 Å, y = 78.8°) in a P1 cell, i.e. without symmetry restrictions, and geometry optimized with the forcefield COMPASS II applying the Ewald summation method until the non-bonding energy was strongly negative.

In case of **Agg2**, equation S3 can be used to calculate the volume of the unit cell:

$$V_\mathrm{unit\,cell} = a * b * c. \tag{S3}$$

Accordingly, four molecules fill the unit cell with a reasonable density of ρ = 1.11 g cm^{-1}.

For the structure of **Agg2** the unit cells with plane symmetry have been considered. Owing to the indexation according to a centered rectangular cell with four PBI molecules (a = 14.1 Å, b = 18.2 Å, c = 27.1 Å) we evaluated first the centered unit cells cm and c2mm. The unit cell should consist of two P- and M-atropo-enantiomers, respectively. However, since exciton theory indicates that neighbors are shifted by 5 Å along the a-axis (for details see main article and ESI section 14), which is less than half of the unit cell, the symmetry breaks and centered plane groups must be excluded. For symmetry reasons most of the rectangular planar groups

can be eliminated. Thus only the monoclinic p1, p2 and the rectangular pg cell remains as possible unit cells. Consequently, the apparent extinction of reflections with h + k = 2n + 1 is accidental and is caused by the specific arrangement of the PBI strands. For steric reasons shifted PBI strands can interact via their π-surfaces only when *M*- and *P*-strands are direct neighbors. Accordingly, a possible unit cell was constructed with the *P*-strands alternatingly shifted by –5 Å and 5 Å with respect to the *M*-strands. The alternating shift is required to prohibit a reduction of the *b*-parameter due to symmetry reasons. This arrangement does not possess symmetry elements and is consequently a p1 unit cell, which however, afforded the best results for the simulated XRS pattern (Figure S13b). Applying geometry optimization at semi-empirical PM7 level of theory,[S3] the interchromophoric arrangement (longitudinal shift of 5 Å) between the P and M chromophores is retained (Fig. S12b)

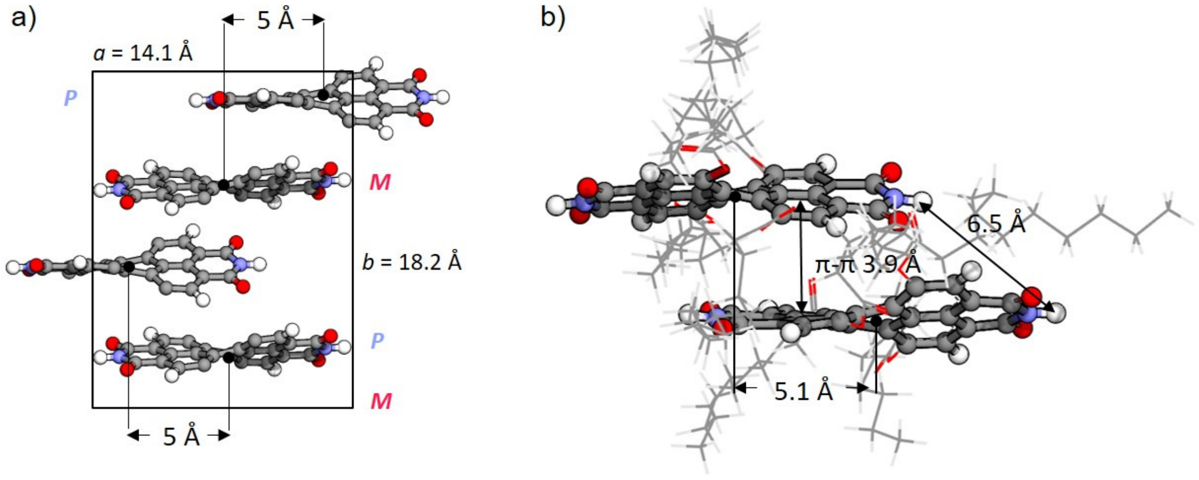

Figure S12. a) View on the *a,b*-plane of the constructed unit cell of **Agg2** containing four PBI molecules. Bay-substituents were omitted for clarity. b) P/M dimer extracted from geometry optimized unit cell (PM7)[S3] with relevant intermolecular distances. Acyloxy-substituents are depicted as lines for clarity. Semi-empirical structure optimization has been performed in MOPAC2016[S4] based on the constructed unit cell given in a) using periodic boundary conditions.

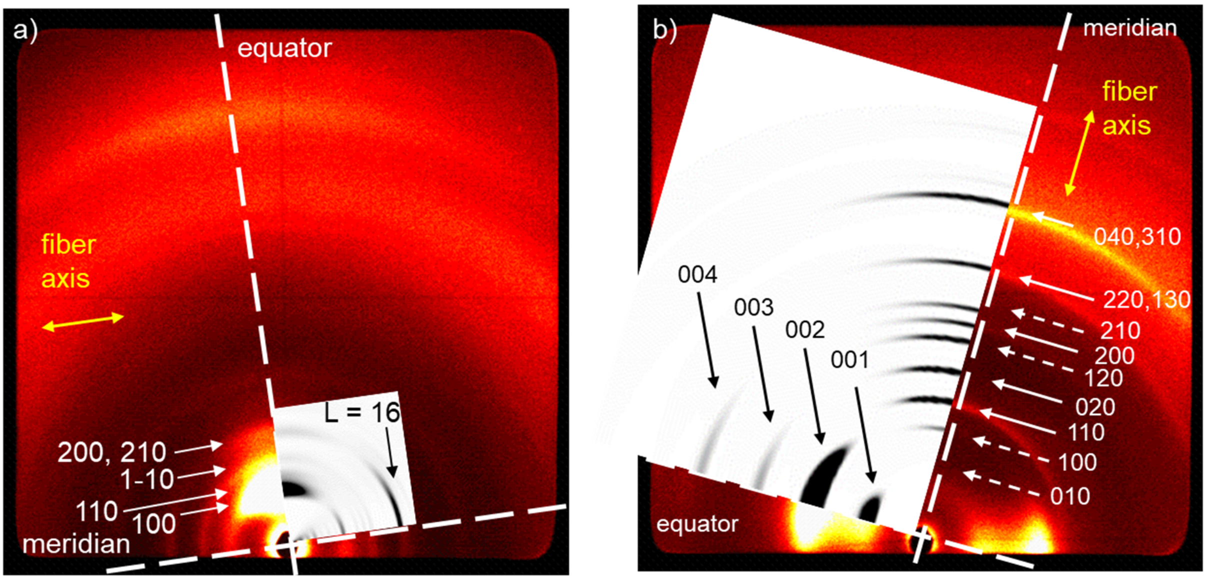

Figure S13. Superposition of the WAXS patterns of a) **Agg1** and b) **Agg2** and the simulated diffraction patterns calculated using CLEARER and the models generated with Materials Studio. The yellow arrows indicate the orientation of the fibers.

The fiber diffraction patterns were simulated with the program CLEARER[S5] using the modelled structures obtained in Materials Studio.[S6]

For **Agg1**, the structure was exported as pdb-file and imported into the fiber diffraction simulation module of CLEARER. The fiber axis was set to (0,0,1) with a crystallite size of *a* = 40 nm, *b* = 40 nm and *c* = 120 nm. The fiber disorder parameters σ_ϕ and σ_θ were set to infinity and 0.5, respectively.

For **Agg2**, the unit cell was exported- as pdb-file and imported into the fiber diffraction simulation module. The fiber axis was set to (0,0,1) with a crystallite size of *a* = 120 nm, *b* = 120 nm and *c* = 40 nm. The fiber disorder parameters σ_ϕ and σ_θ were set to infinity and 0.5, respectively. The crystallite size and contrast were adjusted to best fit the experimental pattern. Meridional and equatorial signals were simulated separately and superposed for illustration. As the constructed unit cell is not inherently centered due to requirements of the longitudinal shift as determined with exciton-vibrational spectral pattern analysis, signals corresponding to a simple rectangular cell appear (dashed arrows in Figure S13b). However, the most prominent reflections (010, 100) are either absent or very weak. The XRS simulation of a soft phase with conformational disordered mesogens is challenging, and a perfect match is difficult to obtain. The present results, however, support that the given model is a good approximation.

13. X-ray scattering

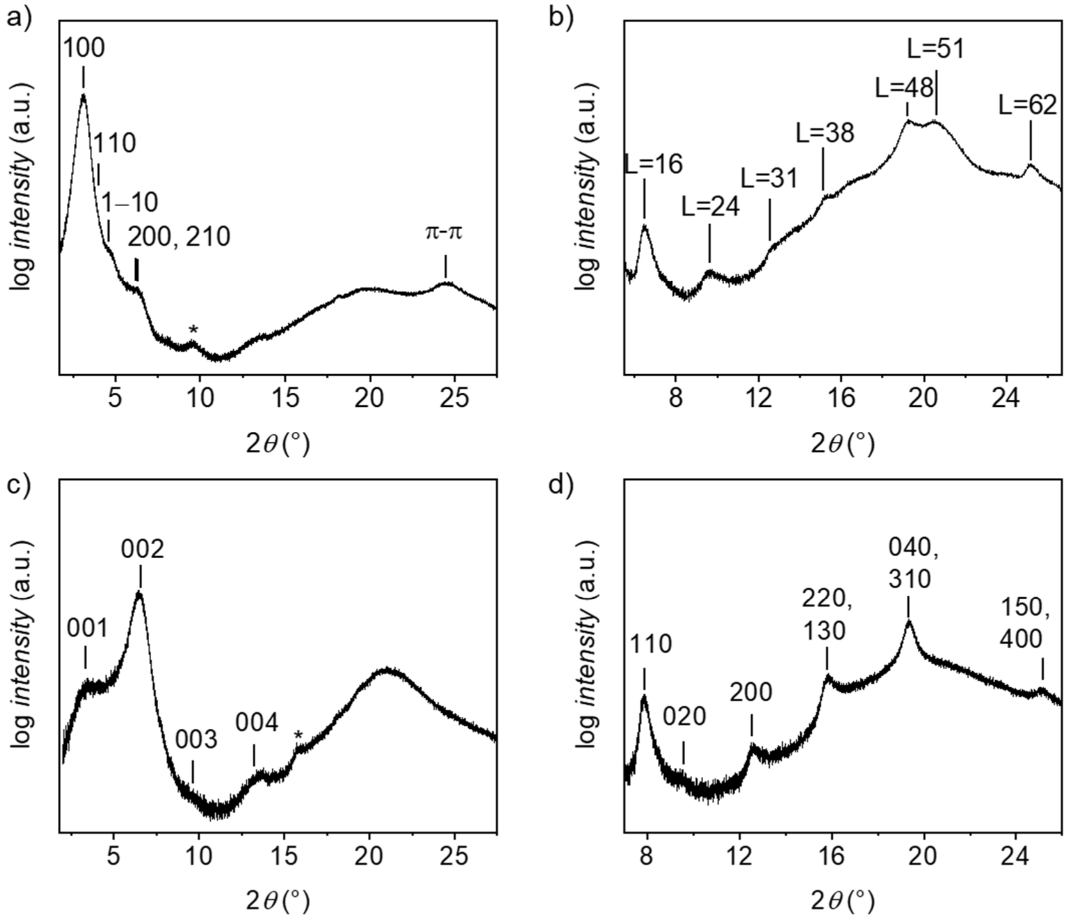

Figure S14. Integrated intensity of the WAXS patterns of **Agg1** (a, b) and **Agg2** (c, d) at 25 °C along the equator (a, c) and along the meridian (b, d). Reflexes indicated with * correspond to residual off-meridional signals.

The correlation length ξ in the LC state can be calculated according to the Scherrer equation[S7]:

$$\xi = \frac{K*\lambda}{\Delta(2\theta)*\cos(\theta)}, \quad \text{(S4)}$$

where K is the dimensionless shape factor with a typical value of 0.9, λ the X-ray wavelength, $\Delta(2\theta)$ the full width at half maximum (FWHM) in radians and θ the Bragg angle in degrees.

For **Agg1**, we calculated the correlation length for the π-stacked PBIs perpendicular to the columnar direction by this method. $\Delta(2\theta)$ and θ were determined by fitting the signal on the equator belonging to the π–π stacking distance. The number of correlated molecules *N* was then calculated by dividing the correlation length by the π-distance. Accordingly, a correlation length of 21.5 Å was determined. This correlates to 21.5 Å/3.6 Å = 6.0 molecules and hints towards the formation of a six-stranded arrangement. This is in agreement with the density considerations described above. Furthermore, the number of correlated columns was calculated using the 100 reflection. The correlation length could be determined as 117.0 Å.

Accordingly, the number of correlated columns equates to 117.0 Å/28.8 Å = 4.1 columns. This weak correlation causes the broadness of the observed signals.

For **Agg2**, we calculated the correlation length along the *c*-axis. The number of correlated lamellae was then calculated by dividing the correlation length by the *c*-parameter. Accordingly, a correlation length of 49.2 Å could be determined. This correlates to 49.2 Å/27.1 Å = 1.8 lamellae demonstrating the weak correlation along the *c*-axis. As the individual lamellae (2D sheets) grow under thermodynamic conditions from solution and only arrange in the lamellar lattice in the solid state, the weak correlation is expected.

14. Comparison to calculated spectra

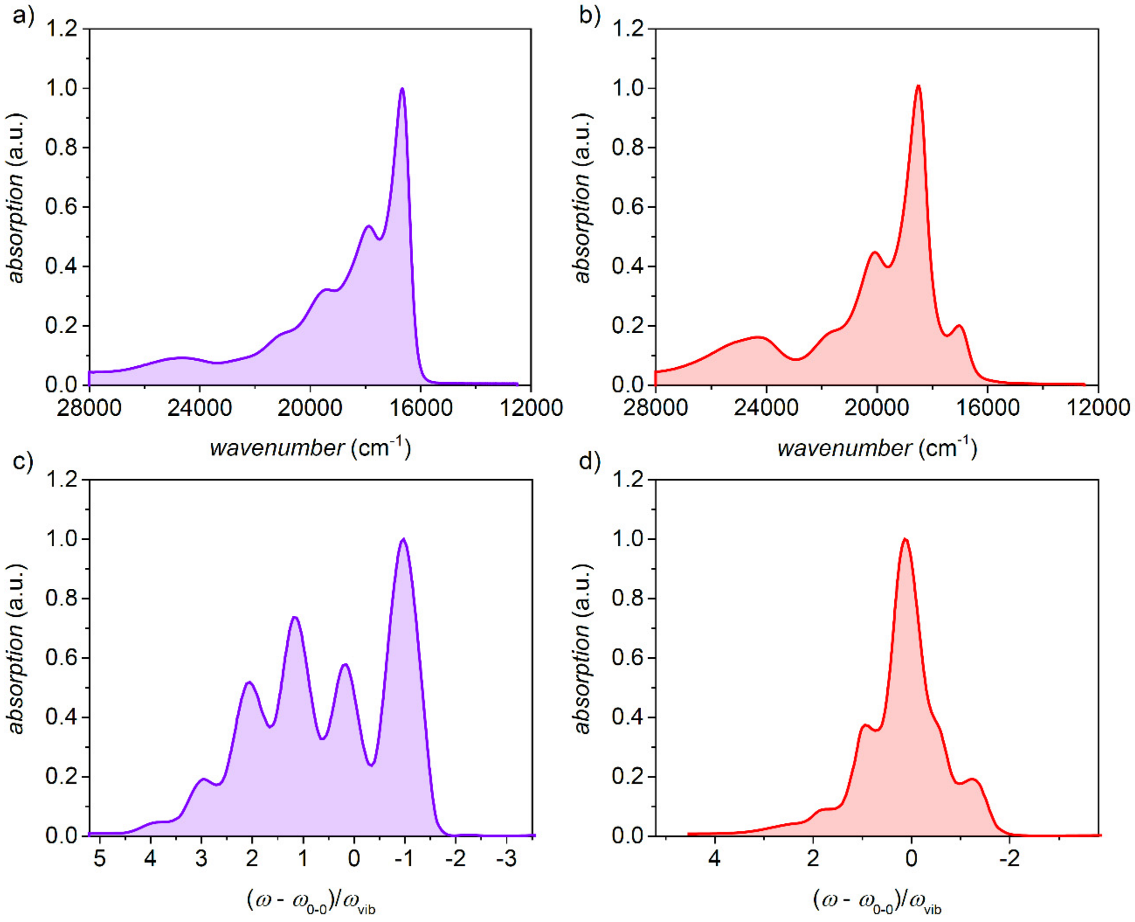

Figure S15. Experimental UV/Vis absorption spectra of a) **Agg1** and b) **Agg2** (c_0 = 40 µM, MCH, 20 °C) and calculated absorption spectra by Hestand and Spano for longitudinal slips of c) 8.0 Å and d) 5.0 Å for a π-stack containing N = 10 perylene molecules.[S8]

XY data of the calculated spectra was extracted from Reference[S8] using WebPlotDigitizer Version 4.2 (San Francisco, USA). In this work, the authors set the diabatic CT energy resonant with the molecular excitation energy $E_{CT}–E_{S1}$ = 0. Please note: Spectra shown in Figure S15 were calculated for perylenes, not PBIs. However, Since the HOMO and LUMO distribution of perylene[S9] and perylene bismide[S10] chromophores are almost identical, we can use the perylene spectra as reference.[S8] The absorption at ca. 400 nm (25000 cm^{-1}) in the experimental spectra is due to the S_0-S_2 transition which is not considered in the calculated spectra.

The spectra were calculated for 1D decamers of perylenes that exhibit short-range coupling only to one perylene on each side of the π-surface.[S8] In our case, the PBIs interact with one further chromophore that is longitudinally displaced as the PBIs form H-bonded strands. In case of **Agg1**, this can be neglected as both chromophore on each side of the π-surface are shifted by ca. 7 Å causing the spectral features typical for PBI J-aggregates[S10-14] in which both,

short- and long-range coupling are of J-type.[S8] In case of **Agg2**, a longitudinal shift between 3.8 Å and 6.3 Å leads to H-type contributions of both, short-range and long-range coupling, that causes the distinctive absorption spectrum shown in Figure S15d.[S8] A longitudinal shift of 5 Å yielded the best result for retrostructural analysis (Figure S13b). Accordingly, a second chromophore on each side of the π-surface is longitudinally displaced by ca. 9 Å (Figure S16). However, as the long-range coupling scales with ~r^{-3} within the point-dipole approximation, where *r* is the center-to-center distance between the coupled transition dipoles, and the short-range coupling decreases exponentially with increasing distance,[S15] the contribution of the second chromophore on each side that is longitudinally shifted by longer distances can be neglected.

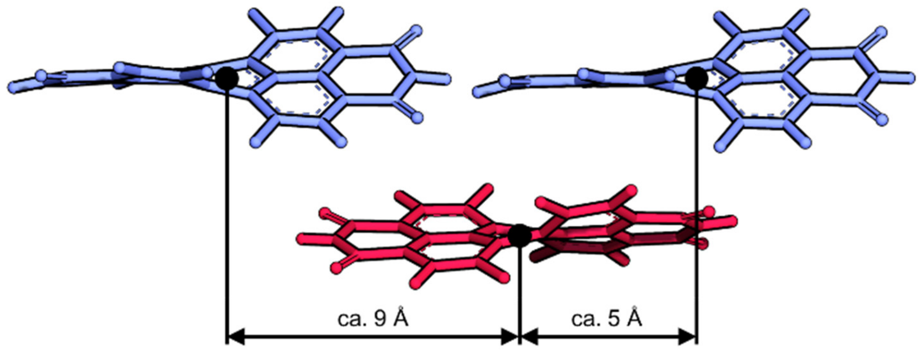

Figure S16. Illustration of the different longitudinal shifts between *M*- (red) and *P*-atropo-enantiomers (blue) in **Agg2**.

15. Temperature-dependent UV/Vis absorption studies

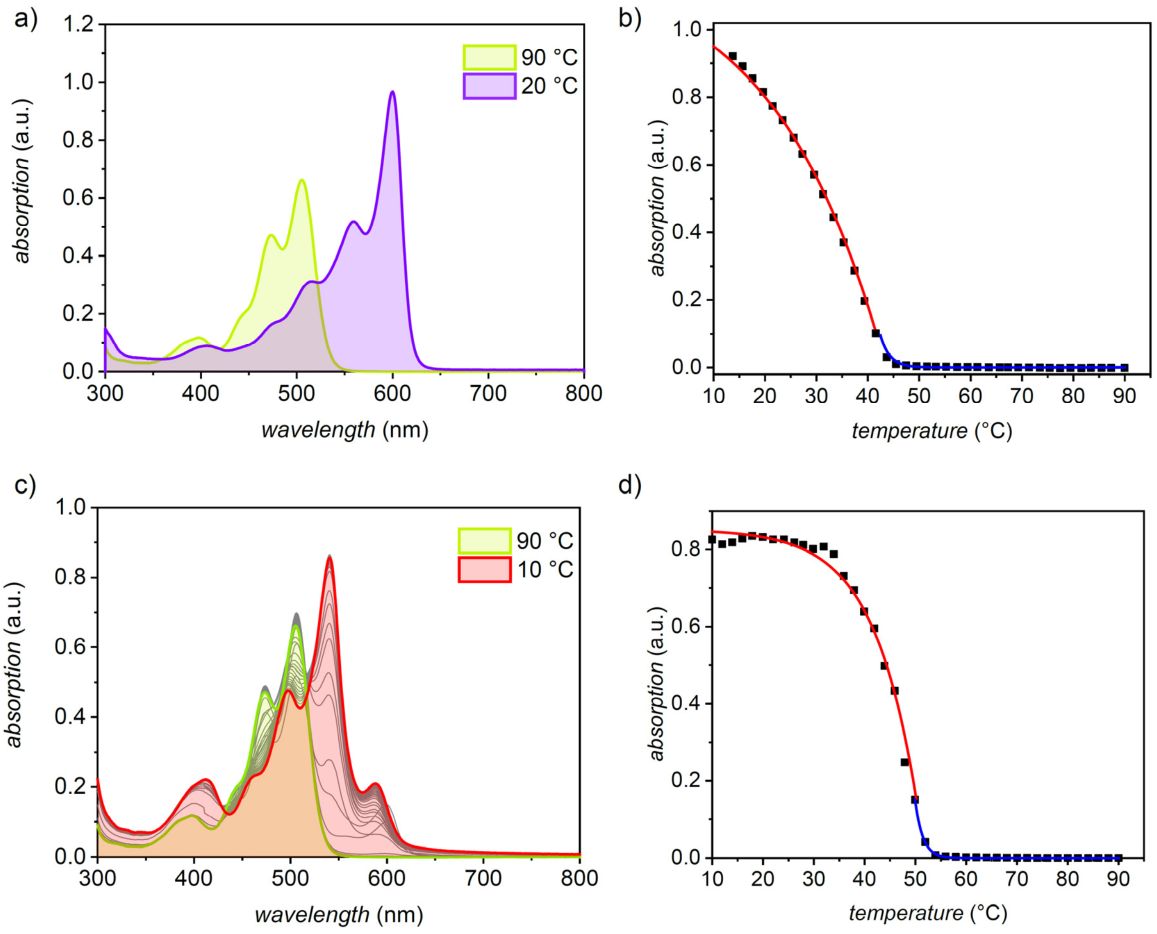

Figure S17. a) Temperature-dependent UV/Vis absorption spectra of **PBI1** with c_0 = 40 µM in methylcyclohexane starting from a monomer solution (green) and applying a) a cooling rate of 10 K/min to direct self-assembly into **Agg1** (purple) and c) cooling rate of 0.6 K/min to direct self-assembly into **Agg2** (red). Plot of the corresponding absorbance of b) **Agg1** at 600 nm and d) **Agg2** at 541 nm as a function of the temperature and fit according to the cooperative nucleation-elongation model[S16-S18] (elongation in red, nucleation in blue).

The change in absorption upon self-assembly of both, **Agg1** and **Agg2** could be fitted with the cooperative nucleation-elongation model.[S16-S18] For **Agg1** an enthalpy of $\Delta H = -39$ kJ mol^{-1}, an elongation temperature of 44 °C and a value for the dimensionless equilibrium constant of the activation step $K_a = 1.5 \times 10^{-4}$ were obtained (c_0 = 40 µM, MCH). For **Agg2** an enthalpy of $\Delta H = -103$ kJ mol^{-1}, an elongation temperature of 52 °C and $K_a = 1.0 \times 10^{-3}$ were obtained (c_0 = 40 µM, MCH).

16. Time-dependent UV/Vis spectroscopy

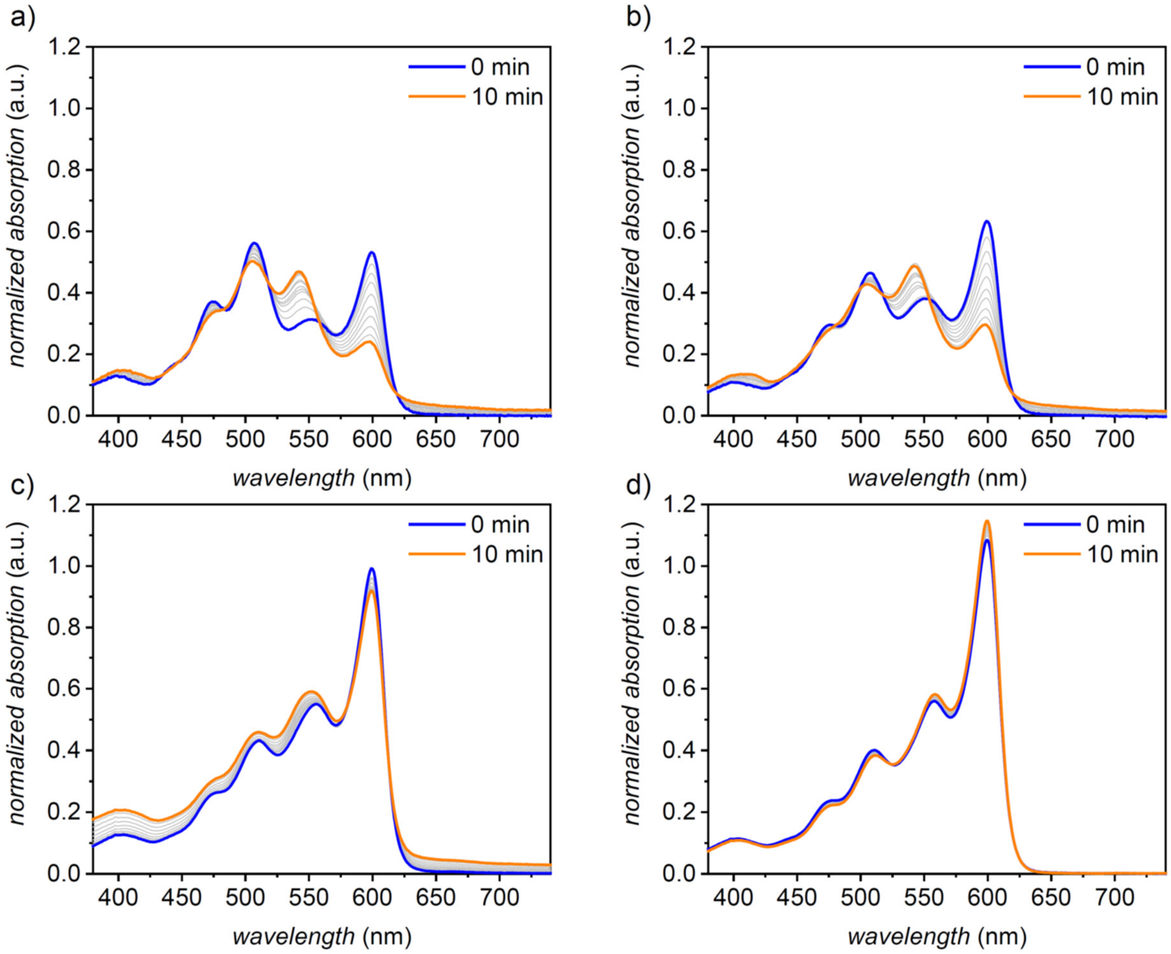

Figure S18. a) Time-dependent UV/Vis absorption spectra of **Agg1** at a) c_0 = 20 µM, b) c_0 = 30 µM. c) c_0 = 50 µM and d) c_0 = 60 µM at 35 °C. At lower concentrations **PBI1** is not fully aggregated, as indicated by the absorption signals corresponding to monomeric **PBI1**.

For time-dependent experiments, the sample solutions were heated to 90 °C for 20 minutes to ensure a fully monomeric state. After rapidly cooling to 35 °C (15 K/min) to produce **Agg1**, UV/Vis absorption measurements were started immediately and spectra were recorded in one minute intervals to monitor the transformation of **Agg1** into **Agg2**.

17. Seed-induced supramolecular polymerization

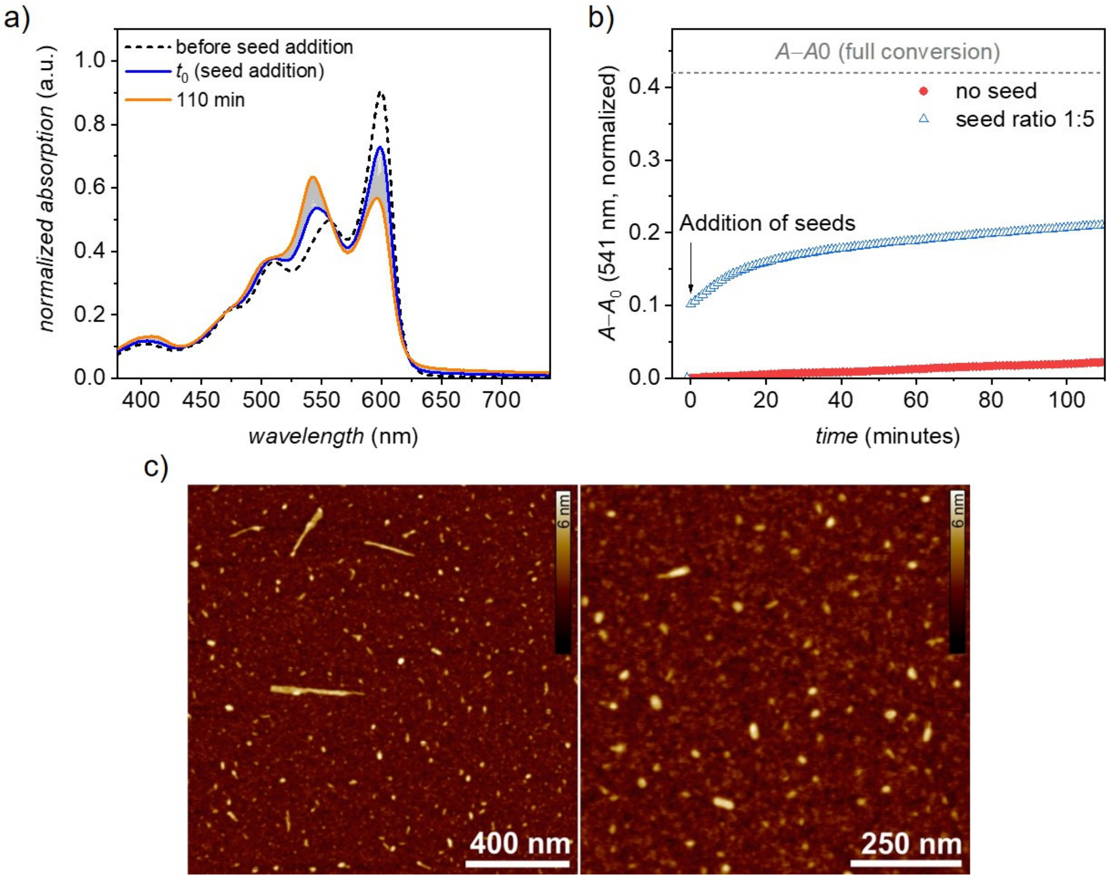

Figure S19. a) Time-dependent UV/Vis absorption spectra for the transformation from **Agg1** into **Agg2** (c_T = 60µM) at 35 °C after addition of seeds of **Agg2** at a **Agg2**$_{\textbf{seed}}$:**Agg1** ratio of 1:5. b) Time course of the transformation from **Agg1** to **Agg2** at 35 °C after the addition of seeds of **Agg2** at a **Agg2**$_{\textbf{seed}}$:**Agg1** ratio of 1:5 (blue triangles) and without the addition of seeds (red dots). c) AFM images of seeds of **Agg2** prepared by ultrasonication of **Agg2** in MCH (*c* = 60µM) for 30 minutes and spin-coating the dispersion on silicon wafer (2000 rpm).

Seeds of **Agg2** were prepared by treating a solution of **Agg2** in MCH (*c* = 60µM) with ultrasound for 30 minutes. AFM revealed the high quality of the nanoparticle seeds with sizes between 15-400 nm. Upon the addition of seeds of **Agg2** to a solution of **Agg1** (*c* = 60µM, MCH, 35 °C, **Agg2**$_{\textbf{seed}}$:**Agg1** ratio of 1:5), enhanced transformation of **Agg1** into **Agg2** can be observed compared to a solution of **Agg1** without seed. However, despite the high seed quality and a high amount of seed, only a slightly improved conversion of **Agg1** can be detected.

18. NMR spectra of PBI1

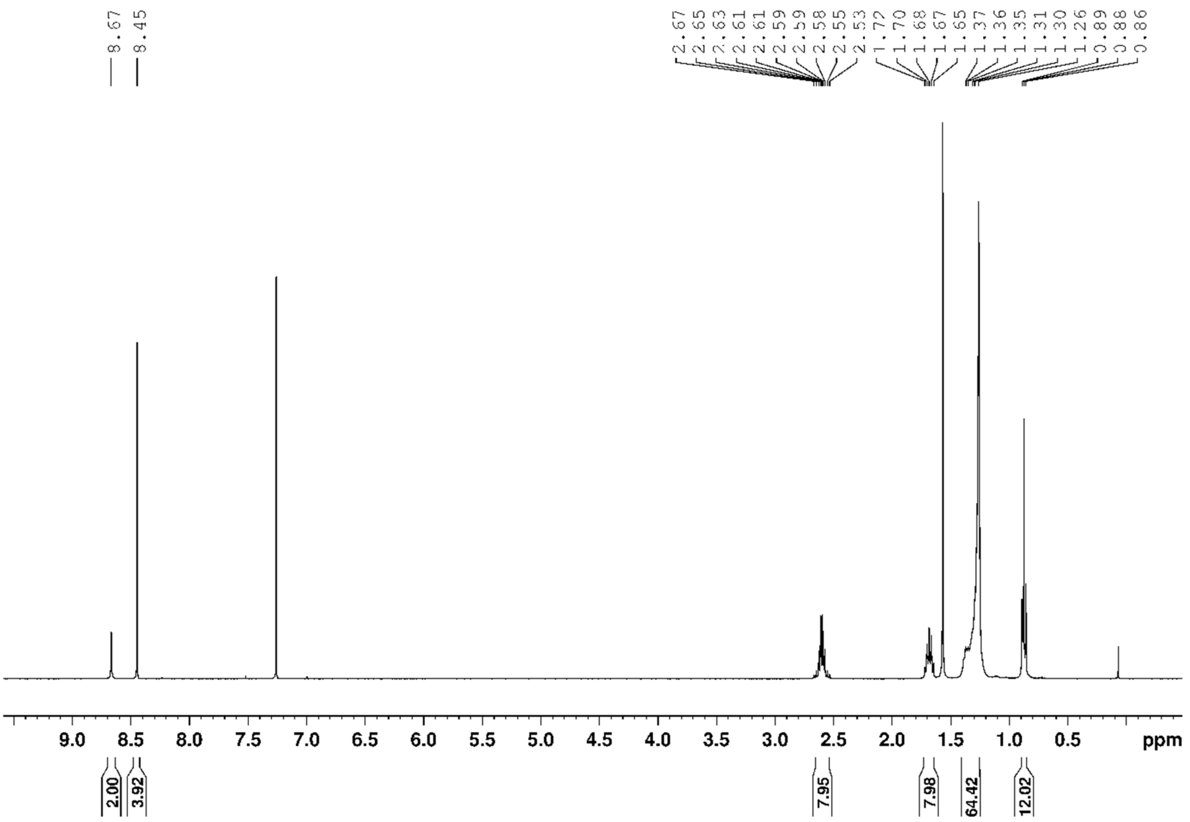

Figure S20. ^{1}H NMR ($CDCl_3$, 400 MHz, 295 K) of **PBI1**.

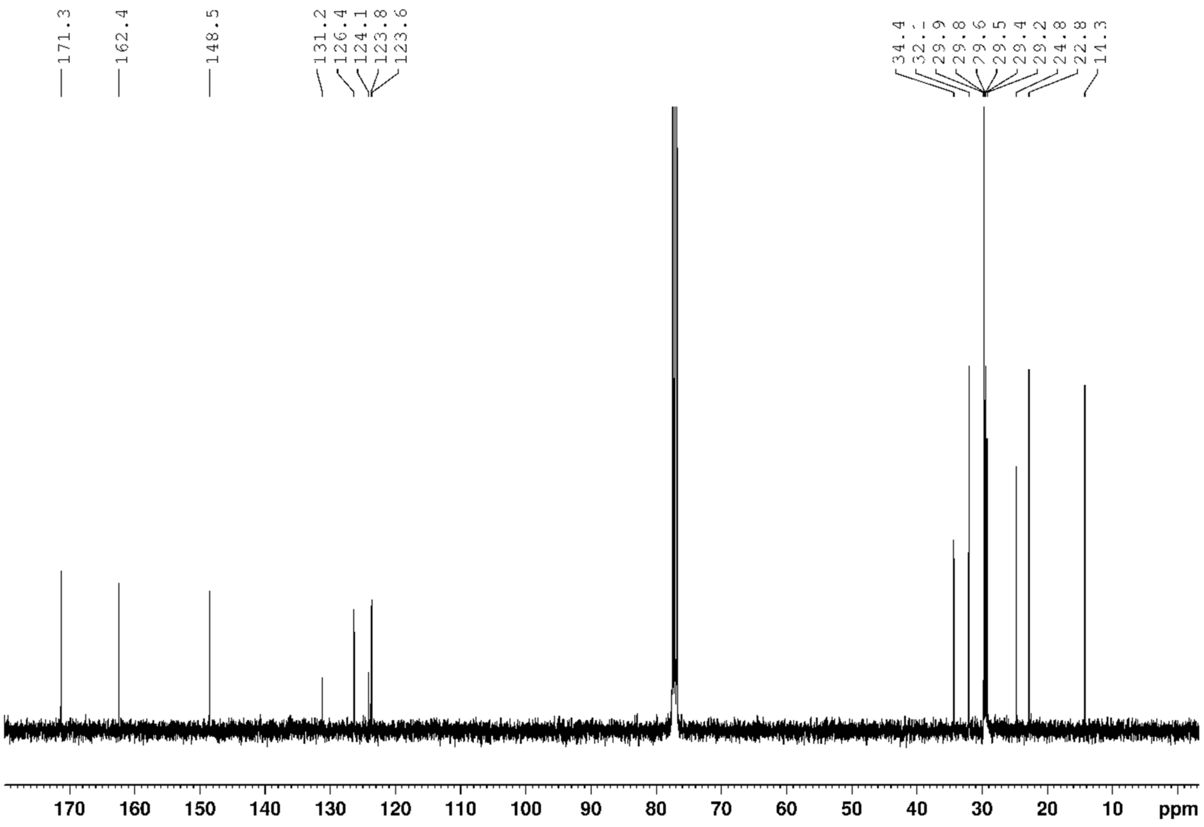

Figure S21. ^{13}C NMR ($CDCl_3$, 100 MHz, 295 K) of **PBI1**.

19. Supporting References

[S1] G. Seybold, G. Wagenblast, *Dyes Pigm.* **1989**, *11*, 303–317.

[S2] C. M. Cardona, W. Li, A. E. Kaifer, D. Stockdale, G. C. Bazan, *Adv. Mater.* **2011**, *23*, 2367–2371.

[S3] J. J. P. Stewart, *J. Mol. Model.* **2013**, *19*, 1-32.

[S4] MOPAC2016, J. J. P. Stewart, Stewart Computational Chemistry, Colorado Springs, CO, USA, HTTP://OpenMOPAC.net (2016)

[S5] O. S. Makin, P. Sikorski, L. C. Serpell, *J. Appl. Crystallogr.* **2007**, *40*, 966–972.

[S6] BIOVIA, Materials Studio 2017, Release 17.1.0.48, San Diego: BIOVIA, **2017**.

[S7] P. Scherrer, *Nachr. Ges. Wiss. Göttingen, Math.-Phys. Kl.* **1918**, *2*, 98–100.

[S8] N. J. Hestand, F. C. Spano, *J. Chem. Phys.* **2015**, *143*, 244707.

[S9] N. J. Hestand, F. C. Spano, *Acc. Chem. Res.* **2017**, *50*, 341–350.

[S10] F. Würthner, C. R. Saha-Möller, B. Fimmel, S. Ogi, P. Leowanawat, D. Schmidt, *Chem. Rev.* **2016**, *116*, 962–1052.

[S11] T. E. Kaiser, H. Wang, V. Stepanenko, F. Würthner, *Angew. Chem. Int. Ed.* **2007**, *46*, 5541–5544.

[S12] T. E. Kaiser, V. Stepanenko, F. Würthner, *J. Am. Chem. Soc.* **2009**, *131*, 6719–6732.

[S13] S. Herbst, B. Soberats, P. Leowanawat, M. Lehmann, F. Würthner, *Angew. Chem. Int. Ed.* **2017**, *56*, 2162–2165.

[S14] S. Herbst, B. Soberats, P. Leowanawat, M. Stolte, M. Lehmann, F. Würthner, *Nat. Commun.* **2018**, *9*, 2646.

[S15] G. D. Scholes, K. P. Ghiggino, *J. Phys. Chem.* **1994**, *98*, 4580–4590.

[S16] M. M. Smulders, M. M. Nieuwenhuizen, T. F. de Greef, P. van der Schoot, A. P. Schenning, E. W. Meijer, *Chem. Eur. J.* **2010**, *16*, 362-367.

[S17] T. F. A. De Greef, M. M. J. Smulders, M. Wolffs, A. P. H. J. Schenning, R. P. Sijbesma, E. W. Meijer, *Chem. Rev.* **2009**, *109*, 5687-5754.

[S18] M. M. Smulders, A. P. Schenning, E. W. Meijer, *J. Am. Chem. Soc.* **2008**, *130*, 606-611.
